# Use of convalescent plasma in pregnant women with early stage COVID-19 infection in a tertiary care hospital in Dubai, February to March 2021: a case series study

**DOI:** 10.1186/s12884-022-05043-w

**Published:** 2022-09-25

**Authors:** Heba Adan, Deemah Harb, Komal Hazari, Widad Abdelkareem, Fareeda Nikhat Khan, Maryam Zouaoui, May Raouf, Doaa Elsawy, Aida Joseph Azar, Amar Hassan Khamis, Abeer Ammar

**Affiliations:** 1 Latifa Women and Children Hospital, Dubai Health Authority, Dubai, United Arab Emirates; 2 College of Medicine, Mohammed Bin Rashid University of Medicine and Health Sciences (MBRU), Dubai Health Care City, Dubai, United Arab Emirates; 3 Hamdan Bin Mohammed College of Dental Medicine, Mohammed Bin Rashid University of Medicine and Health Sciences (MBRU), Dubai Health Care City, Dubai, United Arab Emirates

**Keywords:** SARS-Cov-2, COVID-19, Convalescent plasma, Pregnancy, Dubai

## Abstract

**Background:**

The use of COVID-19 convalescent plasma (CCP) for the treatment of SARS-CoV-2 infection in pregnancy is intriguing in view of its safety profile in pregnancy and historical precedence of the use of plasma for other viral illnesses. This study aimed to evaluate the use of CCP in pregnant women with early COVID-19 infection.

**Methods:**

This is a retrospective case series study. We have included seven pregnant women admitted with early COVID-19 infection to a tertiary care hospital, Latifa Maternity Hospital in Dubai, United Arab Emirates between 12 February and 04 March 2021 and who consented to receive COVID-19 convalescent plasma as part of their treatment plan. Main outcomes measured were clinical and radiological features, laboratory tests, WHO clinical progression scale pre and post treatment, and maternal, fetal outcomes. COVID-19 clinical severity was classified according to the NIH guidelines for criteria of SARS-CoV-2. For the radiological features, a modified chest X-ray scoring system was used where each lung was divided into 6 zones (3 on each side upper, middle, and lower). Opacities were classified into reticular, ground glass, patchy and dense consolidations patterns.

**Results:**

Seven pregnant women with early COVID-19 were enrolled in this study, their mean age was 28 years (SD 3.6). Four had comorbidities: 2 with diabetes, 1 with asthma, and 1 was obese. Five patients were admitted with a WHO clinical progression score of 4 (hospitalized; with no oxygen therapy) and 2 with a score of 5 (hospitalized; oxygen by mask/nasal prongs). Upon follow up on day 10, 6 patients had a WHO score of 1 or 2 (asymptomatic/mild symptoms) indicating clinical recovery. Adverse reactions were reported in 2 patients, one reported a mild skin rash, and another developed transfusion related circulatory overload. All patients were discharged alive.

**Conclusion:**

CCP seems to be a promising modality of treating COVID-19 infected pregnant women. However, further studies are needed to ascertain the efficacy of CCP in preventing progressive disease in the management of COVID-19 infection in pregnant women.

## Introduction

The pharmaceutical management of COVID-19 pregnant women poses a dilemma to obstetricians in terms of maternal and fetal safety profiles of the currently available medication regimens for COVID-19 infections.

Coronavirus infection 2019 caused by the severe acute respiratory syndrome coronavirus 2 (SARS-CoV-2) was declared a global pandemic in early 2020 by the World Health Organization (WHO) [1] and since then has led to a wide range of clinical presentations and loss of lives [2].

Pregnant women are especially considered a high-risk group when compared to the general population in view of the physiological immune modulation and changes in pulmonary function associated with pregnancy itself [3]. Among pregnant women with COVID-19 infection, a systematic review of 192 studies showed that they are more likely to have severe disease when compared to non-pregnant population. Around 11% of the 64,000 pregnant women included in the systematic review had severe disease, 3.3% needed ICU admission, 1.6% required invasive ventilation, 0.11% required ECMO and 0.8% of them died [4].

Despite the higher risk of disease severity in pregnant women, currently available pharmaceutical regimens for COVID-19 infection poses a dilemma to obstetricians in terms of their unknown maternal and fetal safety profiles.

Convalescent plasma has been used since the 1918 flu epidemic, 2015 SARS epidemic and more recently during the MERS (Middle East Respiratory virus) and the Ebola virus epidemics [5] and is of significant interest in the current COVID-19 pandemic. It seems to be a safe and effective treatment strategy in pregnancy since current antiviral or immunomodulatory therapies as of now lack definite safety profile in pregnancy [5–8].

This is one of the few studies on the favorable outcome of the early use of convalescent plasma (CCP) in pregnant women with moderate COVID-19. We describe seven pregnant women with early COVID-19 infection treated with COVID-19 convalescent plasma (CCP) early rather than later in the course of the illness aiming to prevent further progression and complications in this vulnerable group. Adequate randomized controlled clinical trials are required to further study the efficacy of this modality of treatment as standard of care for early COVID-19 infection in pregnancy.

## Methods

### Aim

This study aims to evaluate the use of covid-19 convalescent plasma as a treatment option in pregnant women with moderate covid-19 infection early in the course of the disease.

### Study design

This is a retrospective case series study.

### Setting

This study was conducted in a tertiary care hospital, the Latifa Women and Children Hospital in Dubai, United Arab Emirates between 12 February and 04 March 2021. During the COVID-19 pandemic, this public maternity hospital was the only hospital in Dubai that accepted pregnant COVID-19 infected patients.

### Participants

Seven pregnant women that were admitted with moderate disease [9], with confirmed COVID-19 infection by nasal PCR testing and onset of symptoms within 1-5 days of presentation were included in this study. Testing for SARS-CoV-2 anti S1 and S2 IgG antibody titers was done for all cases in the study subjects to ensure negative levels. A value below 12 AU/ml was considered negative with testing using DiaSorin Liason XL using chemiluminescence immunoassay (CLIA) technology [10]. All patients received standard treatment for COVID-19 infection as per the hospital policy and were counselled for convalescent plasma therapy.

### Study method

A data collection tool was formulated including clinical and radiological features, laboratory tests, maternal and fetal outcomes and WHO disease severity score pre and post treatment [11]. We adopted the Clinical severity criteria of SARS-CoV-2 infection as per the NIH COVID-19 treatment guidelines to classify disease severity of the patients [12]. For the radiological features, we used a modified chest X-ray scoring system in which each lung was divided into 6 zones: 3 on each side upper, middle, and lower. Opacities were classified into reticular, ground glass, patchy and dense consolidations patterns [13]. The patients were rounded on daily by the medical and obstetric team; and chest X-rays and labs were repeated every 72 hours. Furthermore, after discharge patients had telephone follow-up on days 10 and day 28 post-infection.

Collection of plasma from recovered COVID-19 patients was approved by Dubai Scientific Research Ethics Committee (DSREC) of Dubai Health Authority (DHA) as part of treatment for COVID-19 infection, including for pregnant patients [14]. CCP units were collected from eligible, voluntary non remunerated donors who donated plasma post recovery from COVID-19 after obtaining their consent. The donor selection for the collection of CCP was done by Dubai blood donation center physicians as per the AABB Toolkit for COVID-19 CCP under the FDA’s emergency use authorization (EUA) [15].

The FDA and AABB had issued at the end of March 2020 their recommendation and guidelines for blood facilities to start manufacturing such products under controlled conditions and recommended health care facilities to evaluate its effectiveness and safety. However, implementation first started on August 23rd 2020 when the FDA issued an Emergency Use Authorization (EUA) for the use of convalescent plasma in the treatment of hospitalized patients with COVID-19. This was later revised in February 2021 to authorize only the use of high titer COVID-19 convalescent plasma for the treatment of hospitalized patients with COVID-19 early in the course of the disease [16, 17].

### Patient consent

All seven pregnant patients with moderate COVID-19 signed a written consent and received 1-2 units of convalescent plasma.

## Case summaries

### Case 1

**Fig. 1:**
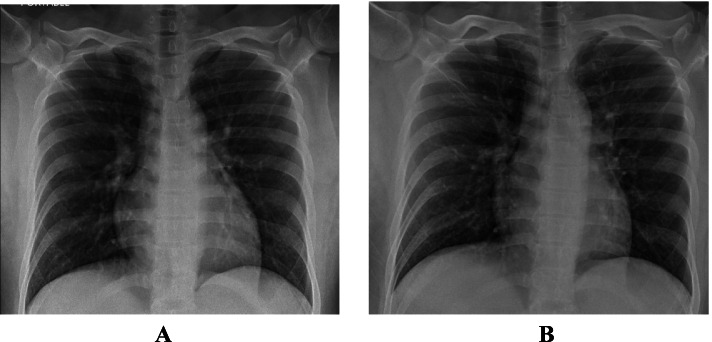
**A** Patient 1 - CXR on admission. **B** Patient 1 - CXR on discharge

Presented with fever, body aches and headache for 2 days. On admission her BP was 123/70 mmHg, respiratory rate of 21-22, O2 saturation of 98% and afebrile but tachycardic with a pulse of 110-120. Chest examination revealed bilateral crepitations in the lung bases. Chest X-ray revealed bilateral perihilar haziness and lower 2 zones reticular infiltrates (Fig. 1A).

She received 2 units CCP. She was improving clinically and was discharged on Day 5. Figure 1B shows the patient’s chest X-ray on discharge.

### Case 2

**Fig. 2:**
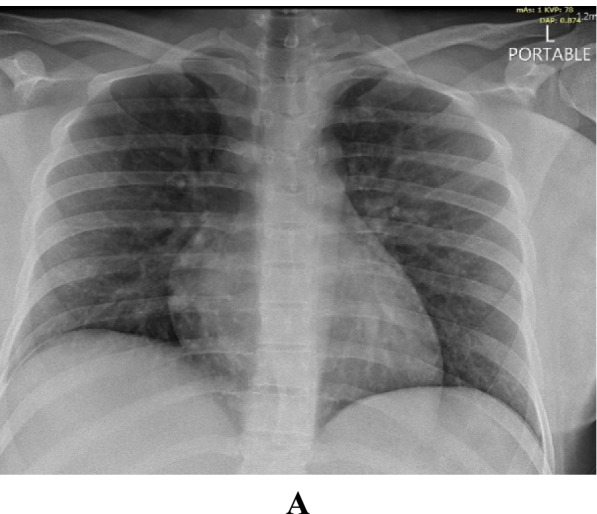
**A** Patient 2 - Chest X-ray on admission

She was admitted with fever, chills, cough, myalgia and loose motion for 2 days duration. Upon admission her BP was 116/75 mmHg, pulse of 100 beats/minute, respiratory rate of 18-20, temperature of 37.8 °C and O2 saturation of 98% on room air. Chest examination revealed bilateral normal air entry with no added sounds. Chest X-ray showed bilateral 4 zones of reticular infiltrates (Fig. 2A).

She was clinically well so discharged on day 5 and repeat chest X-ray was declined by the patient.

### Case 3

**Fig. 3:**
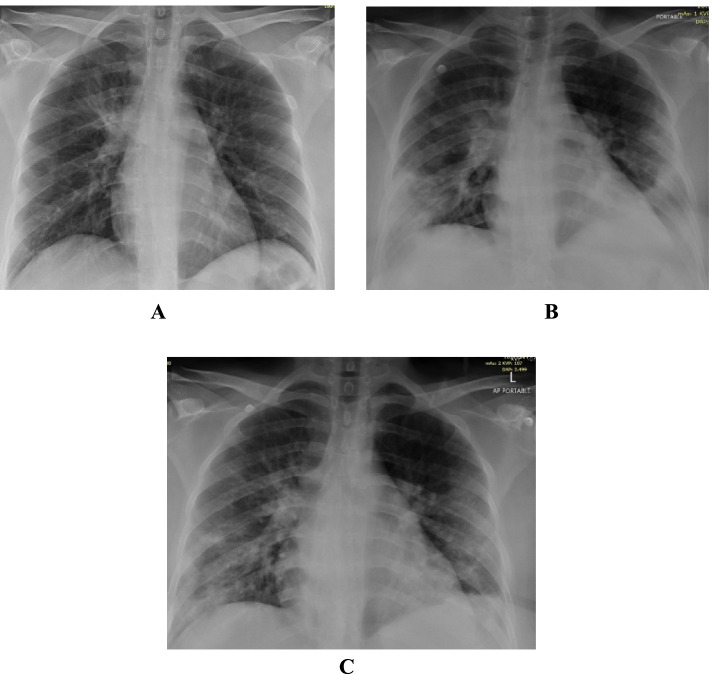
**A** Patient 3 - CXR on admission. **B** Patient 3 - Follow up CXR. **C** Patient 3 - CXR on discharge

She was admitted with fever, sore throat, myalgia and loose motions for 2 days. She looked dehydrated. Her BP was 101/62 mmHg, pulse 110 beats/minute, respiratory rate of 18, temperature of 39.2 °C and an O2 saturation of 97% on room air. Chest examination revealed bilateral basal crepitations. Her chest X-ray showed bilateral 4 zones reticular and ground glass infiltrates (Fig. 3A).

On the night of admission, she complained of breathing difficulty and chest tightness with fever and was found to be having tachycardia - heart rate of 110-120/min & tachypnea 20-24/min. ECG & Cardiac markers were done which were normal. She was started on oxygen supplementation and systemic corticosteroids.

After the first unit of CCP she felt transiently better but after 18 hours again she deteriorated clinically and radiologically (worsening patchy ground glass appearance – Fig. 3B). She was managed with oxygen supplementation and diuretics with a working differential diagnosis of post transfusion associated circulatory overload (TACO) versus worsening covid infection.

Echocardiogram and cardiac markers were normal. She was showing gradual improvement and was discharged in a stable condition on day 8. Figure 3C shows her chest X-ray upon discharge.

### Case 4

**Fig. 4:**
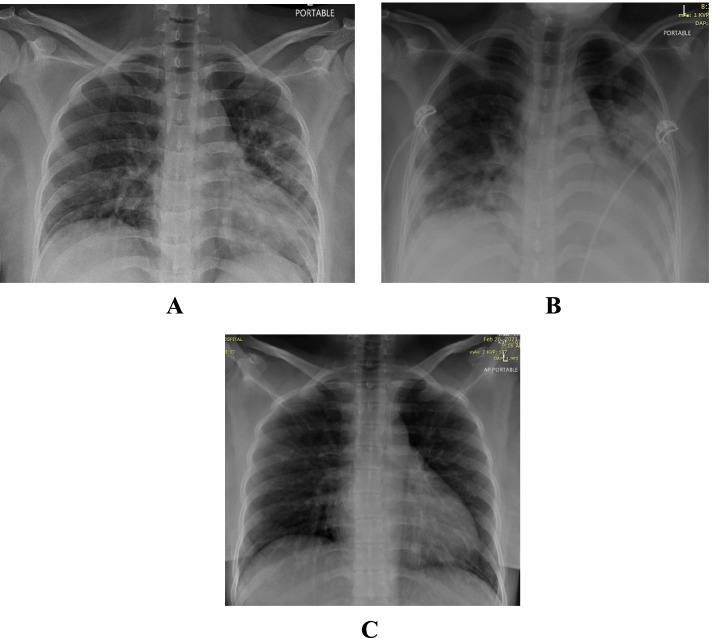
**A** Patient 4 - CXR on admission. **B** Patient 4 - Follow up CXR. **C** Patient 4 - CXR on discharge

She was admitted with symptoms of fever, mild cough, loss of appetite for 4 days and loose stools for 1 day. On admission she had a temperature of 37.9 °C, was tachycardic with HR 122/min, respiratory rate of 18/min and O2 saturation of 94% on room air. Chest examination revealed crepitations over the right base and her X-ray showed ground glass pneumonic infiltrates involving 3 zones (Fig. 4A).

After 16 hours from the 1st unit of CCP she deteriorated clinically and radiologically (Fig. 4B) and hence 2nd unit CCP was withheld. The findings suggested a differential diagnosis of TACO versus worsening COVID-19 pneumonia. She was shifted to ICU and started on NIV and eventually required CPAP. She showed dramatic improvement clinically and radiologically (Fig. 4C) after systemic steroids and Tocilizumab. She was discharged on day 10 in stable medical condition.

### Case 5

**Fig. 5:**
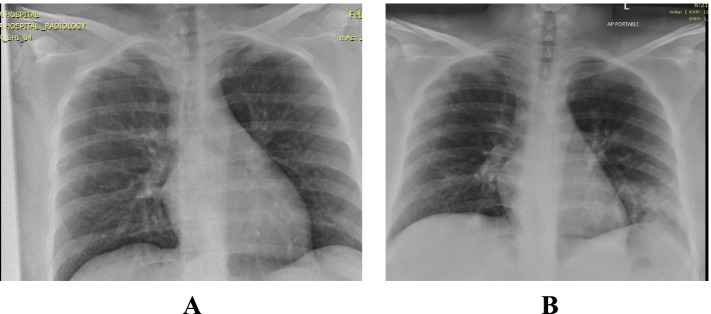
**A** Patient 5 - CXR on admission. **B** Patient 5 -CXR on discharge

Presented to the emergency department with symptoms of fever, cough and diarrhea for 1 day. Clinically she was febrile with a temperature of 38.8 °C; heart rate was 127 beats /min, BP 110/67 mmHg, SpO2 97% and respiratory rate 22/min. Chest examination revealed bilateral basal crepitations. Chest X-ray showed bilateral reticular and ground glass infiltrates involving 3 zones (Fig. 5A).

She consented and received 2 doses of CCP on days 4 and 6 respectively from onset of symptoms without any adverse reactions. She was discharged in good medical condition. Figure 5B shows her chest X-ray on discharge.

### Case 6

**Fig. 6:**
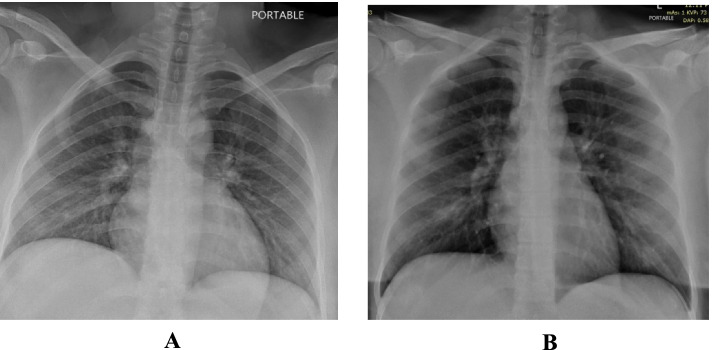
**A** Patient 6 - CXR on admission. **B** Patient 6 - CXR on discharge

Presented with headache, severe myalgia, rhinorrhea and fever. She was vitally stable on admission with BP 121/81 mmHg, pulse of 103, temperature 36.8 °C, respiratory rate of 17/minute and O2 saturation of 99% on room air. Chest examination revealed bilateral normal air entry with no added sounds. Chest X-ray showed bilateral reticular pattern opacities involving 2 zones (Fig. 6A).

She received 2 doses of CCP on day 4 and 5 from symptom onset. She improved clinically and was discharged after 24 hours from the second dose of CCP. Figure 6B shows the patient’s chest X-ray on discharge.

### Case 7

**Fig. 7:**
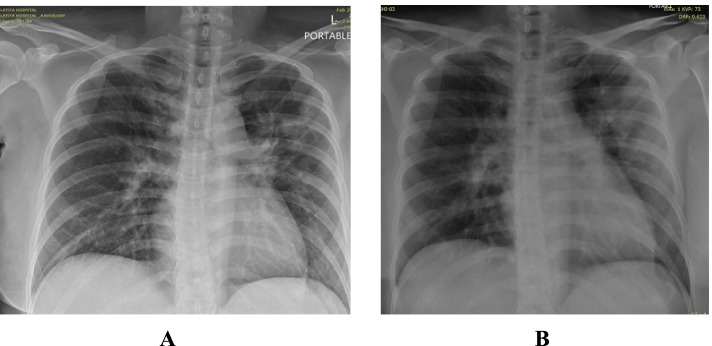
**A** Patient 7 - CXR on admission. **B** Patient 7 - CXR on discharge

She presented with dry cough, body aches, fever and pleuritic chest pain of 1 day duration. Clinically she was afebrile, heart rate of 113 beats /minute, BP 102/56 mmHg, respiratory rate of 18/min and SpO2 of 98% on room air. Chest examination revealed bilateral normal vesicular breathing sounds. Chest X-ray showed 3 zones patchy and ground glass infiltrates (Fig. 7A).

The patient was counselled and agreed for COVID-19 convalescent plasma. She developed a mild skin urticarial rash over the face and thigh region immediately after the completion of the first CCP dose which responded well to IV steroids and antihistamines. Prior to the second dose of CCP, she was pre-medicated with steroids and antihistamine, and she remained well and asymptomatic. She was discharged home on day 3 of admission in stable medical condition. Figure 7B shows the patient’s chest X-ray on discharge.

### Patient involvement

No patients were involved in setting the research question or the outcome measures, nor were they involved in developing plans for design or implementation of the study. No patients were asked to advise on interpretation or writing up of results. There are no plans to disseminate the results of the research to study participants or the relevant patient community.

## Results

Table 1 shows the demographic findings of the 7 hospitalized COVID-19 pregnant women. The mean age was 28 years (SD 3.6), 2 patients were UAE nationals and 5 of different Asian nationalities. The mean BMI was 26 (SD 4.5). The mean gestational age at presentation was 24 weeks (SD 4.6). Four women had comorbidities: one had gestational diabetes mellitus (DM), one had bronchial asthma, one had type 2 DM, and one was obese (Table 1).

**Table 1:** Demographic data, clinical course, CCP administration days and antibody levels, additional treatments, and maternal/foetal outcomes

	Age	Nationality	BMI	Co-morbidities	Gestational Age	Recipient IgG level (neg - < 12)	CCP units transfused	Day from symptom onset to CCP administration	CCP Ab titre 1st & 2nd unit ^a^	Adverse Reaction	Other medications	Length of stay	Maternal / Foetal comment
**1**	29	Filipino	23	GDM	16	< 3.80	2	Day 4, 5	Low, Low	no	HCQ + Lopinavir/ritonavir, LMWH	4	Full term NVD, alive and well
**2**	25	Yemeni	25	No	29	< 3.80	2	Day 5, 6	Low, Low	no	HCQ + Lopinavir/ritonavir, LMWH	4	Full term NVD, Alive & well
**3**	24	Egyptian	29	Asthma	24	< 3.80	1	Day 2	Low	TACO	Steroids, Bioferon, Lopinavir/ritonavir, LMWH	8	Full term NVD, Alive & well
**4**	27	Indian	22	No	24	< 3.80	1	Day 5	Low	no	Steroids, Remdesivir, LMWH, Bioferon, Tocilizumab	10	Full term CS, Alive & well
**5**	30	UAE	35	Obesity	23	< 3.8	2	Day 5, 6	Low, High	no	Bioferon, LMWH	7	Full term NVD, alive and well
**6**	29	UAE	25	Type 2 DM	30	6.5	2	Day 5, 6	High, High	no	LMWH	4	Preterm NVD, Alive & well
**7**	35	Filipino	23	No	26	< 3.80	2	Day 4, 6	High, High	Allergic skin rash	Azithromycin, LMWH	5	Missing data

^a^ Donor CCP units antibody titre levels. For qualitative and quantitative test results refer to Table 2 for details

All patient’s baseline SARS-CoV-2 IgG Ab (Quantitative) levels on admission were negative (by DiaSorin LIAISON XL test using CLIA - chemiluminescence immunoassay; negative value is below 12 AU/ml). All the COVID-19 pregnant women received CCP therapy within the first 5 days from onset of symptoms: 4 on day 5, 2 on day 4, and 1 on day 2 (Table 1). Seven out of the initial twelve convalescent plasma units transfused to the patients were of low titer upon examination by our blood bank as indicated both in Tables 1 and 2. Table 2 specifically shows the numerical covid IgG antibody titer levels of all the plasma units transfused to the study subjects using two laboratory tests which are used in our facility.

**Table 2:** Convalescent plasma units titer levels

Case no.	CCP date	COV-2 IgG^a^ (Qualitative) ***1st unit***	COV-2IgGII^a^ (Quantitative) ***1st unit***	COV-2 IgG^a^ (Qualitative) ***2nd unit***	COV-2IgGII^a^ (Quantitative) ***2nd unit***
1	15/02/21 & 16/02/21	2.4	44.9 AU/mL	0.13	131.8 AU/mL
2	14/02/2021 & 15/02/2021	1.4	416.8 AU/mL	0.02	1.1 AU/mL
3	18/02/21 & 19/02/2021	1.9	579.1 AU/mL	Received only 1 unit	Received only 1 unit
4	17/02/2021	2.3	355.4 AU/mL	Received only 1 unit	Received only 1 unit
5	24/02/2021 & 26/02/2021	2.3	355.4 AU/ml	5.98	2726.9 AU/mL
6	26/02/2021 & 27/02/2021	5.29	1828.6 AU/mL	6.15	14,960.8 AU/mL
7	01/03/2021 & 03/03/2021	5.26	12,276.4 AU/mL	5.26	12,276.4 AU/mL

^a^ FDA high titre on qualitative Index (S/C) ≥ 4.5 and on quantitative test ≥840 AU/mL [17]

Post CCP transfusion adverse reactions were reported in 2 out of 7 cases: one developed possible TACO (transfusion related circulatory overload) but clinically there was a suspicion of worsening COVID-19 pneumonia, and one developed a mild skin rash (Table 1).

The mean total days of hospitalization was 6 days (SD 2.38). Only one patient with a relatively more severe disease was hospitalized for 10 days (Table 1). All patients were discharged alive.

**Fig. 8:**
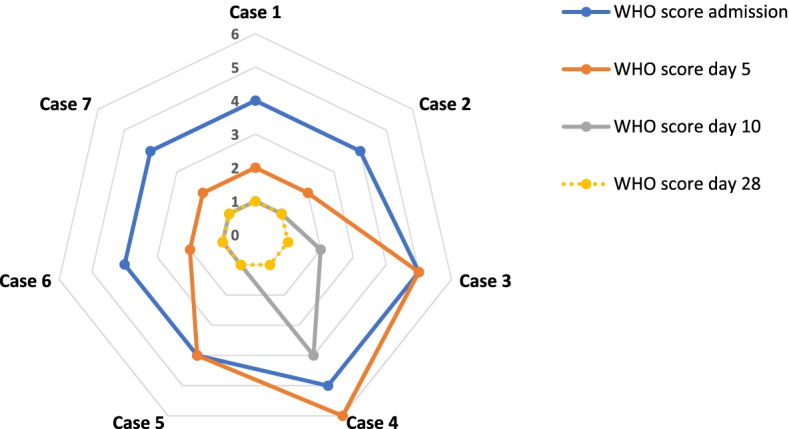
Progression of illness as per WHO scores for each case

All the 7 COVID-19 pregnant hospitalized patients showed clinical improvement as assessed by the change in their WHO clinical progression scale scoring (Fig. 8). On admission, 5 patients had a WHO score of 4 (hospitalized; with no oxygen therapy) and 2 had a WHO score of 5 (hospitalized; oxygen by mask or nasal prongs) (Fig. 8). Upon follow up on day 10, 6 patients had a WHO score of 1 or 2 (asymptomatic or mild symptoms with no need for any assistance); and only one patient who initially presented more severely, still showed a WHO score of 4 (hospitalized; with no oxygen therapy) (Fig. 8). On day 28, all 7 patients had WHO score of 1 (asymptomatic) (Fig. 8).

Five out of the 7 women had term deliveries: 4 by vaginal and 1 by Lower segment Cesarean section (LSCS) (Table 1). One woman had preterm vaginal delivery due to induction of labor at 35 weeks for very uncontrolled sugars in a private facility. Data for 1 patient is missing due to inability to contact her (Table 1).

**Fig. 9:**
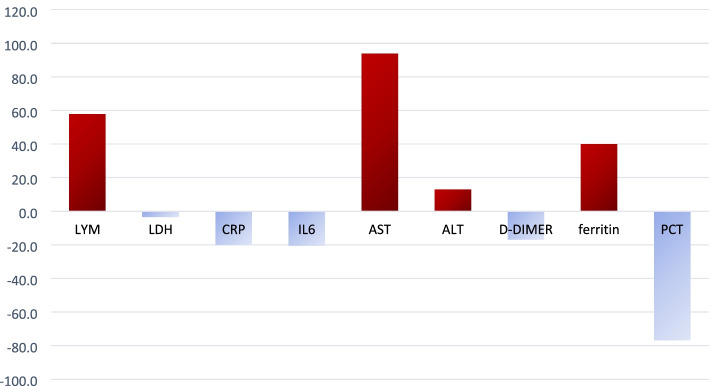
Trend of change in lab parameters with time

The absolute lymphocyte count showed a positive response with a rate of change by 57.8%, ferritin by 39.9% while LDH, CRP, IL-6, Procalcitonin and D-dimer showed a decline in values as depicted in Fig. 9. The liver enzymes showed a positive rate of change of almost 93% for AST and 12.9% for ALT and these are expected to take time after a viral illness to return to normal.

## Discussion

The first 5 cases in the series received convalescent plasma units which were considered of low titer as per the cutoff levels of the different testing modalities used by the blood bank in our facility (Table 2). In the early phase of treatment with convalescent plasma, plasma units were taken from previously infected individuals with positive antibody titers regardless of the exact antibody titer level. Later, the FDA updated guideline on the use of only high titer convalescent plasma came on 11th February 2021 as it was deemed more efficient and hence Dubai Blood Donation Center started releasing convalescent plasma units as per FDA guidelines after 24th February 2021 [18]. Therefore, patients who received COVID-19 convalescent plasma after this date in our facility had received units with high antibody titers.

The concept behind this modality of treatment is that this plasma contains a higher dose of immunoglobulins against the SARS-CoV-2 virus and this provides passive immunity to patients affected by this virus. Normally, patients affected by COVID-19 infection may take 2-3 weeks to develop an adequate antibody response. Hence, providing these antibodies to patients early in the course of the disease aids in neutralizing the virus, decreasing its entry into cells and theoretically enhances more rapid elimination of the virus [19].

Pregnancy is a unique clinical situation where even though they are a higher risk population, the current pharmaceutical interventions, most of which are still experimental, pose a dilemma to use for both clinicians and the patients in terms of their safety profile for the mother and developing fetus.

Convalescent plasma is a blood byproduct and has been widely used before in pregnancy when needed. Good quality evidence on CCP safety and efficacy is lacking so far in COVID-19 infected pregnant women. However, in other viruses such as varicella zoster virus and rabies virus, immunoglobulins have been used in pregnancy to prevent illness progression in these infections with established maternal and fetal safety [12].

Recently, a systematic literature review was done which is the first of its kind to study convalescent plasma for pregnant women with COVID-19 infection. It found only 12 out of 79 records were relevant and these were mostly case reports on 12 pregnant patients. Despite its significant limitations and poor study quality, the conclusion was that the literature seems to indicate that the use of convalescent plasma even for severe cases of COVID-19 infection in pregnant women proves beneficial to both mothers and fetuses [9].

Initially convalescent plasma was thought to be of use in severe COVID-19 infection which would usually be late in the course of the disease. Since then, studies in the non-pregnant population have pointed that this route of management may not prove effective because by then organ damage has started through the now understood acute inflammatory response associated with COVID-19 infection [20]. Consequently, researchers put forward a hypothesis; what if the convalescent plasma was given early in the course of the disease before the onset of the more severe signs and symptoms, to give the body the defense mechanism to fight the virus off [8, 9, 11]. This seemed like a promising idea worth investigating and even more so in pregnant women as plasma components have been shown to be used safely and widely before in pregnancy [21]. In addition to the presumed benefit of giving the convalescent plasma earlier in the course of the disease, it was our observation that patients with less than 3 zones lung involvement and relatively milder chest X-ray findings i.e. with reticular or ground glass patterns had a more favorable outcomes in comparison to those with more than 3 zones lung involvement and patchy or dense consolidations.

The documented associated risks are low and include allergic reactions, TACO, TRALI, transmission of viral infections such as HIV, HBV, and thrombotic/thromboembolic and cardiac events [22].

In our case series of 7 patients, all were either in the second or third trimester with WHO clinical score of 4 or 5. They had initial low COVID-19 IgG levels and were in their early stage of disease between the second to fifth day of symptom onset. Five cases received 2 units of CCP, while 2 of them received 1 unit of CCP due to the development of TACO in one case and worsening COVID-19 course in another. However, none of the cases progressed to critical illness and all of them were discharged in a stable condition. It was our observation that patients who received CCP with high antibody titers had a more favorable course of illness in terms of the positive change in WHO clinical progression score and length of stay as compared to those who received low titer CCP. Only 1 patient had TACO after the first CCP transfused which is a known adverse effect of plasma products.

We agree that like other immune deficiency states, pregnancy although not a pathologic state, is physiologically associated with a depletion in B-cell immune response rendering our patients more at risk to viral illnesses and their complications. Similar to the findings of Rodionov and colleagues, who studied convalescent plasma in 14 patients with varying immunodeficiency conditions, we anticipate that pregnant women may benefit from convalescent plasma for the same reason [23].

A more recent prospective cohort study on convalescent plasma in pregnant patients came to the conclusion that despite the lack of abundant evidence supporting the use of convalescent plasma for pregnant women with covid-19, current data suggests an improvement in both laboratory and ventilatory parameters [24].

Moreover, another study which aimed to analyze the conflicting data coming from the WHO and FDA regarding covid convalescent plasma concluded that upon reviewing 30 randomized controlled trial, indicators of efficacy point to the use of higher titer plasma units [25]. On the other hand a large systematic review and meta-analysis determined that the use of covid-19 convalescent plasma does not improve disease progression or reduce all-cause mortality [26]. However, it is important to point out that this was irrespective of disease severity and timing of administration. We emphasize again that the best current evidence points to early administration of CCP, for mild to moderate cases and the use of high antibody titer plasma units to achieve maximum benefit for the patient. We do understand however that more studies are needed to establish its efficacy as a standard modality of treatment for covid-19 infection in pregnancy.

## Conclusion

We are amongst the first few to publish a case series on the use of CCP in pregnant women with moderate COVID-19 illness within the first 5 days from onset of symptoms. We started this study even before WHO gave its recommendation to use it early in the disease course in the general population and in our case series has shown a favorable outcome. Our study is however limited by the small sample size and lack of follow up of the IgG antibody levels post CCP treatment.

Nevertheless, adequately powered randomized controlled trials are still needed to prove the efficiency of this modality of treatment and whether we can suggest this treatment as standard of care for early COVID-19 infection in pregnancy.

## Data Availability

The dataset analyzed is not publicly available due to the strict regulations of the Dubai Health Authority regarding confidential patients records which does not allow the authors to disclose. However, anonymized data summaries are available by the corresponding authors upon request.

## Abbreviations

BMI: Body mass index
CCP: COVID-19 convalescent plasma
COVID-19: Coronavirus Disease 2019
CS: Cesarean section
DHA: Dubai Health Authority
DM: Diabetes mellitus
HCQ: Hydroxychloroquine
ICU: Intensive care unit
LWCH: Latifa Women and Children Hospital (Dubai)
NVD: Normal vaginal delivery
SARS-CoV-2: Severe Acute Respiratory Syndrome Coronavirus 2
TACO: Transfusion related circulatory overload
UAE: United Arab Emirates
WHO: World Health Organization
